# Plant electrical signals reveal the joint interactions of bicarbonate- selenium on cadmium transport in *Cardamine violifolia*

**DOI:** 10.1080/15592324.2025.2486075

**Published:** 2025-03-31

**Authors:** Juyue Xiao, Antong Xia, Yanyou Wu, Dapeng Wang, Zhanghui Qin, Jiqian Xiang, Gratien Twagirayezu

**Affiliations:** a College of Forestry, Guizhou University, Guiyang, Guizhou, China; b The Key Laboratory of Environmental Pollution Monitoring and Disease Control, Ministry of Education, School of Public Health, Guizhou Medical University, Guiyang, Guizhou, China; c Key Laboratory of Selenium Biotechnology, Enshi Tujia and Miao Autonomous Prefecture Academy of Agricultural Sciences, Enshi, Hubei, China; d Institute of Geochemistry, Chinese Academy of Sciences, Guiyang, Guizhou, China

**Keywords:** Selenium- cadmium antagonism, bicarbonate, electrophysiological parameters, intracellular nutrient translocation, hyperaccumulator selenium plant species

## Abstract

*Cardamine violifolia* (*C. violifolia*), a hyperaccumulator selenium plant species, is a common medicinal and edible species as the primary source of Se supplementation in karst areas. Bicarbonate (HCO_3_^−^), a byproduct of carbonate rock weathering, may interact with Se, but the synergistic effects of HCO_3_^−^ and Se on Cd transport in selenium hyperaccumulators remain unclear. In this study, *C. violifolia* was used to examine the impact of different bicarbonate levels on its growth, photosynthesis, intracellular water dynamics, and nutrient transport. As one result, Se^6+^ improved the intracellular water-holding capacity (IWHC), the intracellular water/nutrient transfer rate (WTR/NTR), the nutrient translocation capacity (NTC), the nutrient active translocation capacity (NAC), while simultaneously reducing Cd^2+^ translocation. Bicarbonate and Se^6+^ together affected Cd^2+^ transport in *C. violifolia*. The BSC1 treatment (1 mm HCO_3_^−^ addition, 0.46 mm Se^6+^ and 0.27 mm Cd^2+^) maximized biomass and photosynthesis, likely due to low HCO_3_^−^ aiding Se^6+^ translocation and reducing Cd^2+^ movement. Conversely, BSC3 (15 mm HCO_3_^−^ addition, 0.46 mm Se^6+^ and 0.27 mm Cd^2+^) resulted in the smallest biomass and photosynthesis in *C. violifolia*, as the high HCO_3_^−^ level inhibited the translocation of Se^6+^, which decreased the IWHC, WTR(NTR), NTC and NAC. No significant correlation was found between Se-Cd translocation factors, suggesting that HCO_3_^−^ may not directly affect Cd^2+^ transport but could increase root pH, hindering Cd^2+^ movement from roots to shoots. The 1 mm bicarbonate interacting with selenium can decrease translocation of cadmium and enhance the photosynthesis and growth, thereby enhancing the selenium enrichment capacity and biomass of *C. violifolia* in karst areas.

## 1. Introduction

Selenium (Se) is a beneficial element to plant species and human health. Individuals over 15 years old require a daily intake of 40 µg of Se. Despite reducing the previous tolerable upper intake level (UL) for adult from 300 µg to 255 µg per day, approximately 90% areas globally are deficient in selenium.^1^ The hyperaccumulator selenium plant species represent the primary pathway for Se supplementation within the food chain. The hyperaccumulator selenium plant species resources is of significant importance for the provision of selenium supplementation to human beings.^2^ Enshi, a city in the southwest of Hubei province, China (109°4′48″-109°58′42″E，29°50′33″-30°39′30″N), is a selenium-rich region with a widely selenium hyperaccumulator species. The first hyperaccumulator selenium plant species, *C. violifolia*, which was discovered in Enshi,^3,4^ has been subsequently produced and promoted on a global areas.

However, the selenium-rich geological background of Enshi, attributed the elevated Cd levels in soils and plant species, making high Cd exposing risk in *C. violifolia*.^5^ As we know, Cd is a deleterious heavy metal element with passive effects on human health, including bone pain disorders, through various pathways, including environmental exposure and food chain.^6,7^ Meanwhile, Enshi is also a typical karst area, where HCO_3_^−^ is a product of carbonate rock weathering. Additionally, HCO_3_^−^ may affect Se-Cd transport of plant species.^8^ However, previous studies usually only focused on the effect of Se to Cd transport in plant species, it has not been clarified for joint effects of HCO_3_^−^ and Se to Cd transport in plant species.

Previous studies found that Se inhibits the transport of Cd in some plant species. For example, Se promoted antioxidant system^9^ and the maintenance of chloroplast structure,^10^ as well as the inhibition of Cd uptake in plants.^11,12^ Consequently, the addition of Se can reduce the uptake and translocation of Cd in plants. The inorganic forms of selenium, Se^6+^ in selenates (SeO_4_^2-^) and Se^4+^ in selenites (SeO_3_^2-^), are are primarily forms utilized by plants. Se^6+^ in Selenates have a greater uptake rate and higher bioavailability than Se^4+^ in selenites, making Se^6+^ was the main form of selenium supplementation.^13,14^ Otherwise, it is of greater significance that Se^6+^ and HCO_3_^−^ can coexist in karstic alkaline soils.^15^ It suggests that selenate and HCO₃^−^ may act in a synergistic effect to Cd transport in hyperaccumulator selenium plant species. However, it has not been confirmed in recent research. More importantly, it is well established that nutrient ions were exchanged by active and passive transport on plant cell membranes.^16^ Consequently, we assumed that the intracellular water and nutrient metabolism is the determining factor in ion transport of cell membranes. However, the previous studies have focused on elucidating the characteristics of Se-Cd transport in plants, it has not confirmed for the active and passive transport metabolism of HCO_3_^−^, Se^6+^, and Cd^2+^.

Fortunately, plant electrophysiological dynamic parameters provides a new approach for studying the intracellular nutrient transport in plants, and it has been widely used to investigate active and passive nutrient transport in plant cell metabolism, such as *Matthiola incana* (L.),^17^ *Orychophragmus violaceus* (*Ov*)^18^ and *Brassica napus* L.,^19^ etc. Additionally, the plant electrophysiological parameters have been utilized to elucidate the responses of plants’ mechanisms to various environment. Compared to traditional electrical signals, plant electrophysiological dynamic parameters can not only determining electrical signals, such as resistance (R), impedance (Z), capacitance (C), and inductance (L),^19^ but the dynamics of intracellular water and nutrient transport in plant cells.^20–22^ Furthermore, the dynamics of cellular intracellular water encompass several key aspects: the intracellular water holding capacity (IWHC), the intracellular water use efficiency (IWUE), the intracellular water holding time (IWHT), and the water transfer rate (WTR). The cell nutrient transport characteristics encompass nutrient flux per unit area (UNF), nutrient translocation rate (NTR), nutrient translocation capacity (NTC), active transport flow of nutrient (UAF), and nutrient active translocation capacity (NAC). The electrophysiological dynamic characteristics of plants not only capture the true state of plant growth but also precisely reflect the active and passive processes of water and nutrient transport within plant cells. Consequently, they offer significant insight into the combined effects of HCO_3_^−^ and Se^6+^ on Cd^2+^ transport in hyperaccumulator selenium plant species found in karst regions.

In this study, we selected *C. violifolia* as the experimental material and implemented a range of bicarbonate treatments (1, 5, 15 mm HCO_3_^−^ addition) to investigate the combined effects of HCO_3_^−^ and Se^6+^on Cd^2+^ transport. Based on the characters of growth, photosynthesis, plant’s electrophysiological information intracellular water and nutrient transport characteristics，the Se and Cd transport status, we quantified the active and passive transport characteristics of intracellular nutrients in *C. violifolia* using plant electrophysiological dynamic parameters. We aim to solve two questions, (1) the joint effects of HCO_3_^−^ and Se^6+^ on Cd^2+^ transport in *C. violifolia*; (2) Based on plant electrophysiological dynamic characteristics the active and passive transport of Se^6+^ and Cd^2+^ within *C. violifolia* was revealed under different HCO_3_^−^ addition. Hence, it provides a new insight to clarify the joint interactions of HCO_3_^−^ and Se^6+^ to Cd^2+^ transport in karst areas.

## 2. Materials and methods

### 2.1. Plant materials

The test material was *Cardamine violifolia* (*C. violifolia*), which was selenium-rich horticultural plants, sourced from Enshi in Hubei, China. The seedlings of *C. violifolia* were planted in trays with twelve holes (19 × 15 × 9.5 cm), filled with perlite and vermiculite at a 1:3 ratios, and then watered using a modified Hoagland solution to culture it. The nutrient solution was changed every 3 d, and the seedlings were transplanted after 28 d. Experiments were kept under 25°C, in which humidity was 65%, and light time was 12 h/d. In this study, experiments were organized using 3 plants/pot, 3 pots/group, and 3 groups/treatment for different HCO₃^−^, 0.46 mm Se^6 +^ and 0.27 mm Cd^2+^ treatments, which were cultured for 21 d (Table 1).

**Table 1.** The modified composition of Hoagland nutrient solution.

Macroelement	Quantity of matter (mM)
KNO_3_	6
NH_4_Cl	0.75
NH_4_H_2_PO_4_	0.25
Ca(NO_3_)_2_•4 h_2_O	4
MgSO_4_•7 h_2_O	2
A trace element	
KCl	2
H_3_BO_3_	50
CuSO_4_•5 h_2_O	0.2
ZnSO_4_•7 h_2_O	4
MnSO_4_•4 h_2_O	4
(NH_4_)_6_Mo_7_O_24_•4 h_2_O	0.2
Carnallite	
Fe(Na)EDTA	2

### 2.2. Bicarbonate–selenium–cadmium treatment

The Se^6 +^ and Cd^2 +^ solutions were uniformly sprayed onto the pots, with the Se^6 +^ prepared sodium selenate (Na₂SeO₄) and the Cd^2 +^ prepared from cadmium sulfate (CdSO₄) (Table 2).^23^ Additionally, the bicarbonate (HCO_3_^−^) was prepared from NaHCO₃. The control group received only the Cd^2 +^ solution treatment, and all prepared solutions were administered at the same time each day.

**Table 2.** Treatments of HCO₃^−^ - Se^6 +^ - Cd^2 +^ in the study.

Group	Treatment (mMol/L，mM)	
CK	HCO_3_^−:^ Se^6+:^ Cd^2+^	0 mm: 0 mm: 0.27 mm
SC BSC1 BSC2 BSC3	HCO_3_^−:^ Se^6+:^ Cd^2+^ HCO_3_^−:^ Se^6+:^ Cd^2+^ HCO_3_^−:^ Se^6+:^ Cd^2+^ HCO_3_^−:^ Se^6+:^ Cd^2+^	0 mm: 0.46 mm: 0.27 mm 1 mm: 0.46 mm: 0.27 mm 5 mm: 0.46 mm: 0.27 mm 15 mm: 0.46 mm: 0.27 mm

### 2.3. Biomass

The plants were washed and separated into roots, stems, and leaves. Each group was weighed five times using a precision balance (accuracy, 0.0001 g), and the measurements were recorded to calculate the total weight of each plant part for each treatment group.

### 2.4. Photosynthesis

For quantification of leaf gas exchange parameters, the second and third fully expanded leaves were selected as experimental specimens. Following a 2-hour visible light acclimation period (07:00–09:00 hours), measurements of net photosynthetic rate (*P*_N_，μmol(CO_2_)m^−2^·s^−1^), stomatal conductance (*Gi*, mol·m^−2^·s^−1^), intercellular CO_2_ concentration (*Ci*) and transpiration rate (*E*, mmol m^−2^·s^−1^) were conducted using an LI-6400XT Portable Photosynthesis System (LI-COR, Lincoln, NE, USA) equipped with a red LED light source. The measurement protocol maintained standardized environmental conditions: photosynthetic active radiation of 500 ± 50 μmol·m^−2^·s^−1^, CO₂ concentration of 400 μmol mol^−1^, a flow rate of gas of 500 μmol s^− 1^, leaf temperature of 25°C, and relative humidity maintained at 55 ± 5%. All measurements were systematically performed between 09:00 and 11:00 hours under stable environmental parameters. WUE was calculated according to Equation 1 as follows^24^:

(1) $$\mathrm{WUE}=\frac{P_{N}}{E}$$

### 2.5. Intrinsic electrophysiological parameters of plant leaves

**Figure 1.**
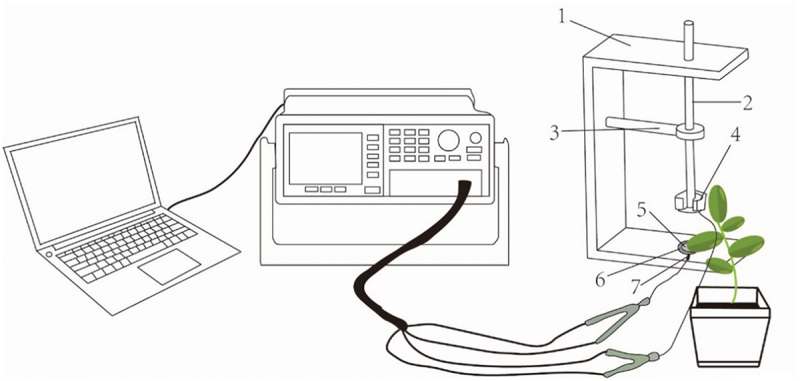
The experimental setup in the study and a schematic diagram of the parallel-plate capacitor [20]. 1= holder; 2= cystosepiment; 3= plate electrode; 4= electrical conductor; 5= iron Block; 6=plastic rod; 7= bench hold.

At the end of the culture period, plants demonstrating consistent growth and overall health under various treatments were carefully selected for testing. The third expanded leaves of plants were placed between the two poles of a parallel plate capacitor, which formed a parallel plate capacitor sensor. With the measurement voltage set at 1.5 volts and the frequency adjusted to 3000 hz. The parallel capacitance sensors were configured in parallel mode for assessment using an LCR tester (LCR-6100, China) (Figure 1). The physiological resistance (R), impedance (Z) and capacitance (C) of the leaves were measured under various clamping force (F), with each force subjected to data collection of fifteen to twenty sets. Fifteen data sets were selected for subsequent calculation and reliable results.

Usually, we considered the chloroplasts of leaves as capacitors, which changed along with the clamping forces, resulting in alterations of electrical signals in plant cells.

According to the law of conservation of energy and the first law of thermodynamics, the work done by the clamping force follows the Gibbs free energy equation, and Gibbs free energy equation can be transformed from dG=dH-TdS to ΔG =ΔH + PV, i.e., dG=ΔG, dH =ΔH. And the energy formula for spherical capacitors is expressed as W = 1/2*C*U^2^. At constant temperature, W represents the energy stored in the capacitor, which is equivalent to the change of Gibbs free energy ΔG, i.e., W = ΔG. ΔH denotes the internal energy of the system (such as a plant leaf system composed of cells), *P* is the pressure exerted on the plant cells, V is the volume of the plant cells, U is the test voltage, and *C* is the physiological capacitance of the plant leaf.

The pressure exerted on the plant cells can be calculated using the equation *p* = F/S, where F is the clamping force, and S is the effective area under the influence of the parallel plate capacitance sensor. Additionally, C of the plant leaves is modeled to change with F.

(2) $$C=\frac{2\Delta H}{U^{2}}+\frac{2V}{SU^{2}}F$$

In formula 2, let $y_{0}=\frac{2\Delta H}{U^{2}}$，$a=\frac{2V}{S{fU}^{2}}$，which can be adjusted into the variation of the C of the plant leaf concerning the F:

(3) $$C=y_{0}+a\;F$$

The capacitive resistance (Xc) was calculated as Equation 4:

(4) $$\mathrm{Xc}=\frac{1}{2\mathrm{\pi f}C}$$

In Equation 4, π is 3.1416, and *f* is 3000 hZ.

Based on R, Z, and Xc, the inductive reactance (X_L_) of plant leaves was analyzed as equations 5

(5) $$\frac{1}{-X_{L}}=\frac{1}{Z}-\frac{1}{R}-\frac{1}{\mathrm{Xc}}$$

Based on the equation of Gibbs free energy and the Nernst, we obtained the models between R (Z/Xc/X_L_) and F is as Equations 6 to 9:

(6) $$R=y_{1}+a_{1}e^{-b_{1}F}$$

(7) $$fZ=y_{2}+a_{2}e^{-b_{2}F}$$

(8) $$\mathrm{Xc}=y_{3}+a_{3}e^{-b_{3}F}$$

(9) $$X_{L}=y_{4}+a_{4}e^{-b_{4}F}$$

When the clamping force (F = 0 N) is applied, the intrinsic resistance (IR), intrinsic impedance (IZ), intrinsic capacitive reactance (IXc), and intrinsic inductive reactance (IX_L_) of plant leaves can be calculated accordingly from Equations 6-9 into Equations 10-13:

(10) $$\mathrm{IR}=y_{1}+a_{1}F$$

(11) $$\mathrm{IZ}=y_{2}+a_{2}F$$

(12) $$\mathrm{IXc}=y_{3}+a_{3}F$$

(13) $$IX_{L}=y_{4}+a_{4}F$$

The intrinsic capacitance (ICP) of plant leaves can be calculated according to Equation 14.

(14) $$\mathrm{ICP}=\frac{1}{2\pi \mathrm{fIXc}}$$

### 2.6. Intracellular water use dynamics

Intracellular water holding capacity (IWHC) of plant leaves was calculated as Equation 15:

(15) $$\mathrm{IWHC}=\sqrt{{\left(\mathrm{ICP}\right)}^{3}}$$

The specific effective thickness (d) of plant leaves was calculated by Equation 16.

(16) $$d=\frac{U^{2}a}{2}$$

The intracellular water use efficiency (IWUE) of plant leaves was calculated using Equation 17.

(17) $$\mathrm{IWUE}=\frac{d}{\mathrm{IWHC}}$$

The intracellular water holding time (IWHT) of plant leaves was calculated using Equation 18.

(18) IWHT=ICP×IZ

The water transfer rate (WTR) of plant leaves was calculated using Equation 19.

(19) $$\mathrm{WTR}=\frac{\mathrm{IWHC}}{\mathrm{IWHT}}$$

### 2.7. Characterization of nutrient translocation capacity

The nutrient flux per unit area (UNF) of plant leaves can be calculated using Equation 20.^25^

UNF

(20) $$\mathrm{UNF}=\frac{p+q}{n}=\frac{\frac{1}{\mathrm{Xc}}+\frac{1}{\mathrm{XL}}}{\frac{1}{R}}\;=\frac{R}{\mathrm{IXc}}+\frac{R}{IX_{L}}$$

‘*p*’ can be characterized as the quantity of protein and lipids that cause the capacitive resistance of biological tissues.

‘*q*’ can be characterized as the quantity of proteins that cause inductive reactance in biological tissues.

Since nutrients are soluble in water, WTR and the nutrient translocation rate (NTR) are conceptually similar and assigned the same value, defined and calculated using Equation 21. Therefore, the nutrient translocation capacity (NTC), is the UNF multiplied by NTR in Equation 22.

(21) NTR=WTR

(22) NTC=UNF×NTR

Consequently, the active transport flow of nutrient (UAF) can be calculated using Equation 23.Therefore, NAC is UAF multiplied by NTR, as indicated in Equation 24.^25^

(23) $$\mathrm{UAF}=\frac{\mathrm{IXc}}{IX_{L}}$$

(24) NAC=UAF×NTR

### 2.8. Se and Cd translocation/bioconcentration factor

After harvesting and drying the samples, the plant samples were digested, and the ICP-MS^26^ was employed to determine the total Se and Cd contents in shoots and roots of *C. violifolia*. Plant samples were digested using an automatic microwave digestion system, and the total Se and total Cd contents were determined by inductively coupled plasma mass spectrometry (ICP-MS). We weighed 0.4000 g of the sample and added 10 mL HNO_3_ and 2 mL H_2_O_2_ to a Teflon digestion tank for 0.5 h. The temperature was increased to 160°C after 10 min, and further raised to 200°C, which was maintained for 0.5 h. After cooling to 25°C, the sample was diluted to 100 mL (ultrapure water) for measurement. The measurement conditions of ICP-MS were set as follows: spectral mode, RF power of 1550W, gas temperature of 2°C, sampling depth of 8 mm, carrier gas flow rate of 0.95 L/min, plasma gas flow rate of 15 L/min, and auxiliary gas flow rate of 0.10 L/min.

Translocation factor (TF) (equation 30) reflects the migration and accumulation of Se^6 +^ and Cd^2 +^ within the soil-plant based on the movement from the soil to the plant roots and subsequently from the roots to the leaves. Bioconcentration factor (BCF) (equation 31) represents the ability of plants to assimilate enriched elements from the soil. It is expressed as the ratio of a specific element concentration in a designated part of the plant to the environmental elements’ concentration as Equations 25 and 26.

(25) $$\mathrm{TF}=\frac{\mathrm{shoot}\left(\mathrm{Se},\mathrm{Cd}\right)}{\mathrm{Root}\left(\mathrm{Se},\mathrm{Cd}\right)}$$

(26) $$\mathrm{BCF}=\frac{\mathrm{Organ}\left(\mathrm{Se},\mathrm{Cd}\right)}{\mathrm{Environ}\left(\mathrm{Se},\mathrm{Cd}\right)}$$

## 3. Results

### 3.1. Growth characteristics of cardamine violifolia

Table 3 illustrates the biomass of roots, stems, leaves, and the overall biomass for *C. violifolia* across different bicarbonate treatments. The biomass of roots, stems, leaves, and the total biomass for *C. violifolia* did not exhibit any significant variations between CK and SC. Conversely, while the biomass of roots, leaves, and the total biomass for *C. violifolia* remained statistically similar between SC and BSC1, BSC1 resulted in a notable increase in stem biomass relative to SC. In contrast, BSC3 led to a significant reduction in the biomass of roots, stems, leaves, and the overall biomass for *C. violifolia* in comparison to SC.

**Table 3.** The biomass of roots, stems, and leaves in *C. violifolia.*

Group	CK	SC	BSC1	BSC2	BSC3
Root FW,g/Plant	2.33 ± 0.44bc	3.00 ± 0.49b	2.74 ± 0.25b	3.82 ± 0.28a	1.73 ± 0.20c
Stem FW,g/Plant	4.83 ± 0.66b	5.07 ± 0.31b	7.74 ± 0.70a	5.19 ± 0.36b	3.04 ± 0.29c
Leaves FW,g/Plant	8.19 ± 1.18a	8.84 ± 0.75a	8.56 ± 1.05a	7.61 ± 0.33a	4.68 ± 0.26b
Total Biomas FW,g/Plant	15.34 ± 1.46b	16.91 ± 1.13ab	19.04 ± 2.00a	16.62 ± 0.94ab	9.45 ± 0.71c

The data are presented as mean ± standard error (M±SE). The same letter means no significant difference in the same line (Duncan’s multiple range tests, *p < 0.01*). Under different bicarbonate treatments, the ratio of Se^6+^/Cd^2+^ in Hoagland solution was 0.46 mm: 0.27 mm.

### 3.2. Photosynthesis

**Figure 2.**
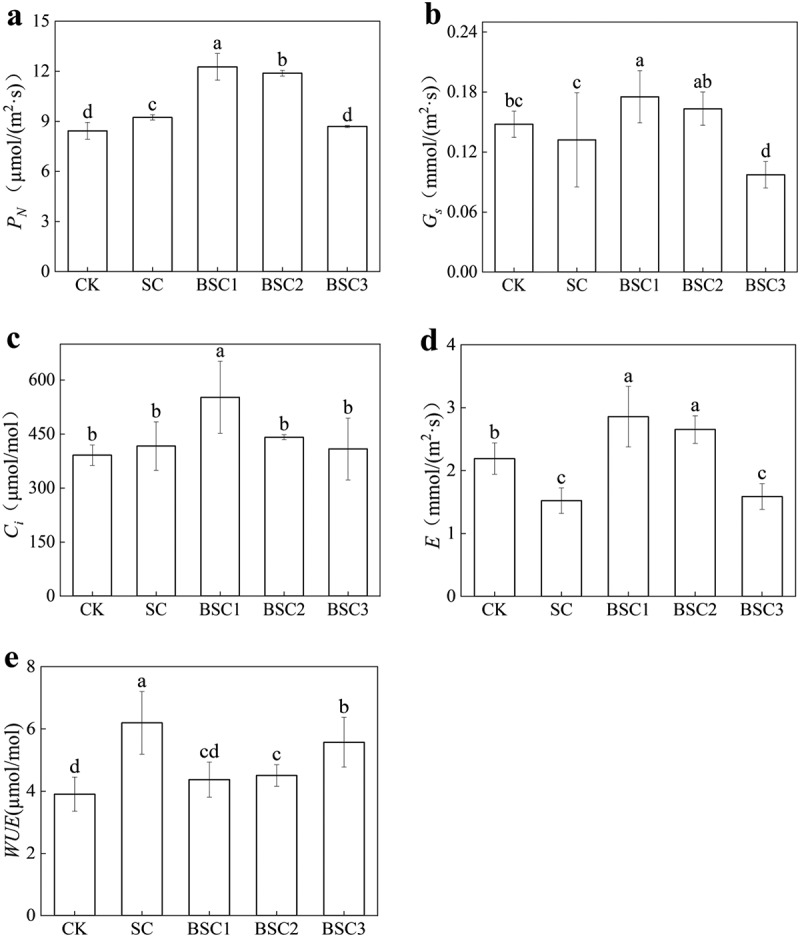
Photosynthetic characteristics of *C. violifolia* under different treatments. (a) net photosynthetic rate (*P*_*N*_，µmol/(m^2^·s))，(b) the pore conductivity (*G*_*S*_， mmol/(m^2^·s))，(c) the intercellular CO_2_ concentration (*C*_*i*_, μmol/mol)，(d) the transpiration rate (*E*， mmol/(m^2^·s)), (e) the water-use efficiency (*WUE*，μmol/mol).

Figure 2 illustrates the effects of different HCO_3_^−^ treatments on the photosynthesis of *C. violifolia*. The net photosynthetic rates (*P*_*N*_, Figure 2a) showed BSC1 > BSC2 > SC > CK (BSC3), with BSC1 exhibiting the most significant increase of 45.43% when compared to CK. The stomatal conductances (*G*_*s*_, Figure 2b) did not exhibit any significant variations between CK and SC. In contrast, BSC1 resulted in a significant increase, whereas BSC3 led to a significant decrease compared to SC. Except BSC1, the intercellular CO_2_ concentrations (*Ci*, Figure 2c) have no significant variations among treatments. Transpiration rates (*E*, Figure 2d) showed BSC1(BSC2) > CK > BSC3(SC), with BSC1 showing a maximum increase of 30.14% over CK. In comparison to CK, SC exhibited a significant enhancement in water use efficiency (*WUE*, Figure 2e). Conversely, bicarbonate resulted in a notable reduction in comparison to SC.

### 3.3. Electrophysiological parameters of the plants

**Figure 3.**
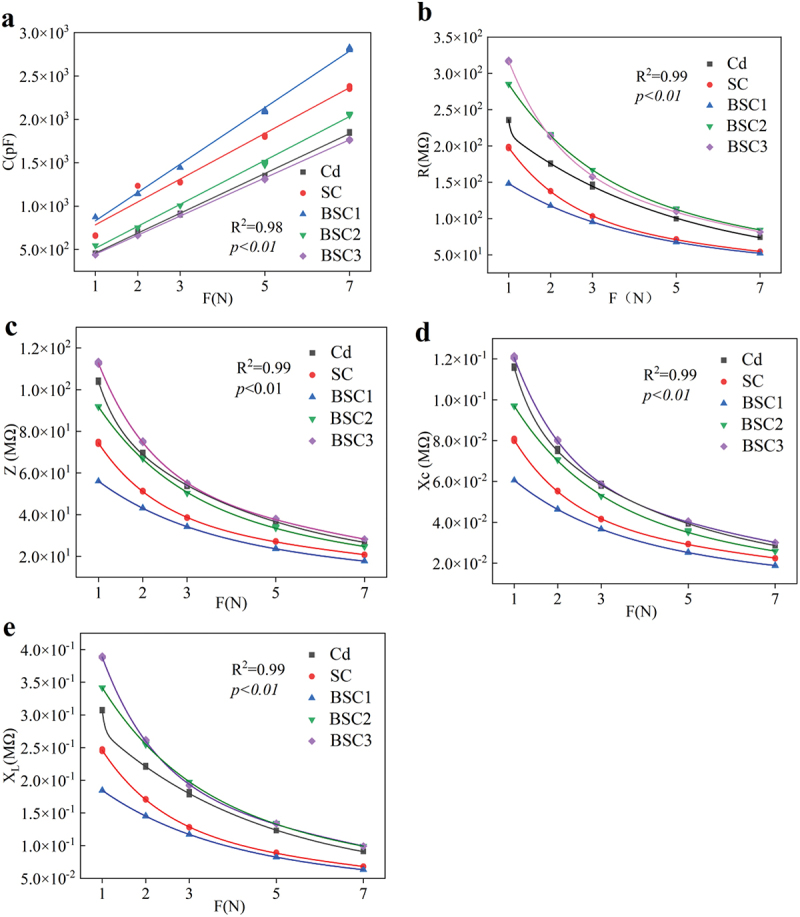
The fitting equations between the electrical parameter and F of randomly selected from the leaves of *C. violifolia* under different treatments. C (a), R (b), Z (c), xc (d), and X_L_ (e) represent capacitance, resistance, impedance, capacitive reactance, and inductive reactance respectively.

C (Figure 3a), R (Figure 3b), Z (Figure 3c), Xc (Figure 3d), and X_L_ (Figure 3e) were randomly selected from the leaves of *C. violifolia* with respect to F. The results indicate a strong correlation between C, R, Z, Xc, and X_L_ with F, with a significance level of *p < 0.01*.

### 3.4. Intrinsic electrophysiological parameters

**Figure 4.**
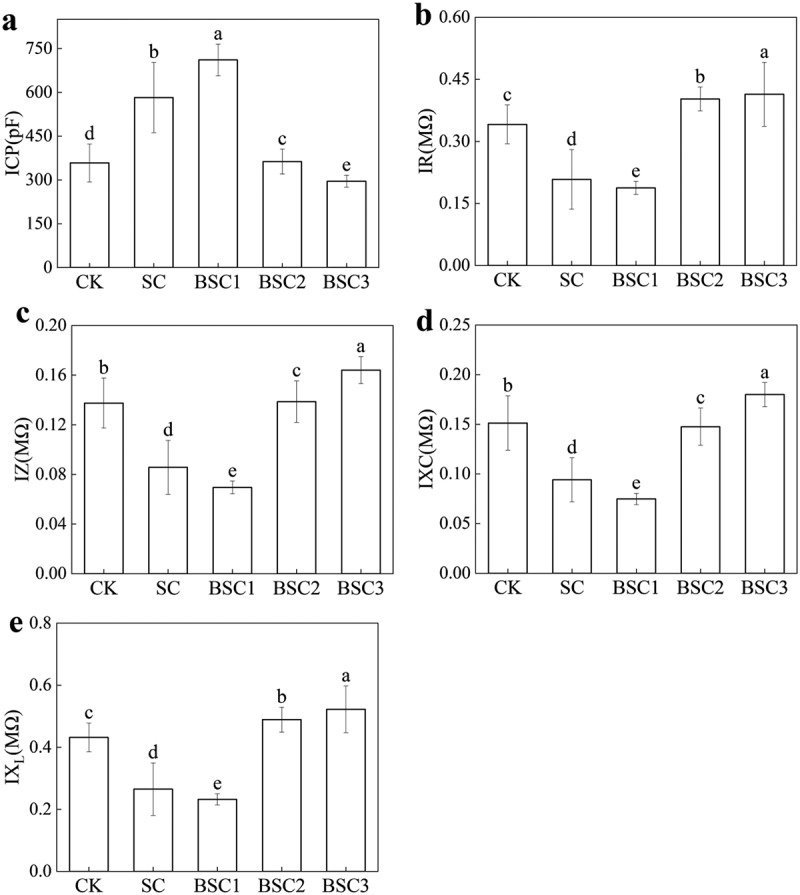
The intrinsic electrophysiological parameters of the plant. The electrophysiological information of *C. violifolia*, (a) ICP represents intrinsic capacitance, (b) IR denotes intrinsic resistance, (c) IZ indicates intrinsic impedance, (d) IXc represents intrinsic capacitive reactance, and (e) IX_L_ denotes inductive reactance.

The intrinsic electrophysiological parameters of *C. violifolia* leaves under different HCO₃^−^ treatments are shown in Figure 4. IR, IZ, IXc, and IX_L_ were greatest in BSC3, and the trends of IZ and IXc consistently as follows: BSC3 > CK > BSC2 > SC > BSC1. Compared to CK, the IZ and IXc of BSC3 obtained maximum increase with 23.08% and 20.00%, respectively, in which the decreased of BSC1 relative to CK was 50.00% and 53.33%. It showed BSC3 > BSC2 > CK > SC > BSC1 of IR and IX_L._ Compared to CK, IR and IX_L_ of BSC3 obtained maximum increase with 20.59% and 20.93%, whereas BSC1 decreased by 47.59% and 46.51%. The ICP showed the opposite trend of the above parameters: BSC1 > SC > BSC2 > CK > BSC3. BSC1 increased by 98,60% compared with CK, while BSC3 decreased by 17.60% compared to CK.

### 3.5. The intracellular water use of *C.* *violifolia*

As represented in Table 4, the intracellular water utilization parameters differed significantly among the five treatments. The IWHC showed a trend with BSC1(SC) > BSC2(CK, BSC3), with a maximum increase of 58.20% in BSC1 compared to CK. IWHT showed no significant difference. WTR showed BSC1 (SC) > CK(BSC2, BSC3), with BSC1 increased by 56.31% compared with CK. IWUE increased with the increase of bicarbonate addition.

**Table 4.** The intracellular water use dynamic parameters in *C. violifolia.*

	IWHC	IWUE(10^−3^)	IWHT	WTR
CK	5033.35 ± 607.86^b^	59.44 ± 3.86^ab^	48.41 ± 1.90^a^	103.81 ± 9.54^b^
SC	6949.06 ± 983.83^a^	45.32 ± 8.42^b^	48.17 ± 0.81^a^	144.50 ± 22.63^a^
BSC1	7962.99 ± 406.07^a^	53.90 ± 6.84^b^	49.20 ± 0.43^a^	161.82 ± 8.04^a^
BSC2	5083.10 ± 406.74^b^	59.26 ± 9.97^ab^	49.84 ± 0.29^a^	101.96 ± 7.60^b^
BSC3	4436.93 ± 203.90^b^	74.37 ± 15.84^a^	48.38 ± 1.76^a^	91.74 ± 4.38^b^

The data are expressed as mean ± standard error (M±SE). The same column means no significant difference in the same line (Duncan’s multiple range tests, *p* < 0.01). The intracellular water metabolism capacity of *C. violifolia*. IWHC denotes intercellular water-holding capacity, IWUE denotes intracellular water-use efficiency, IWHT symbolizes intracellular water-holding time, and WTR represents water translocation rate.

### 3.6. The nutrient translocation capacity of *C.* *violifolia*

Table 5 presents the correlation analysis of nutrient transport parameters in *C. violifolia*. The UNF showed no significant difference. NTR showed a trend same with WTR. In comparison to CK, SC exhibited a significant enhancement in NTC. Conversely, bicarbonate resulted in a notable reduction in comparison to SC. BSC1 resulted in a notable increase in NTC relative to SC. In contrast, BSC3 led to a significant reduction in NTC in comparison to SC. In UAF, it showed no significant difference. NAC showed SC (BSC1) > CK(BSC3, BSC2), with BSC1 increased by 44.01% compared with CK.

**Table 5.** The nutrient transport parameters of leaves in *C. violifolia.*

	UNF (10^−1^)	NTR	NTC	UAF (10^−2^)	NAC
CK	30.95 ± 5.45^a^	103.81 ± 9.54^b^	323.52 ± 77.13^c^	35.18 ± 6.56^a^	36.28 ± 4.88^b^
SC	29.54 ± 2.44^a^	144.50 ± 22.63^a^	423.21 ± 34.77^b^	36.08 ± 2.68^a^	52.53 ± 11.69^a^
BSC1	33.14 ± 1.74^a^	161.82 ± 8.04^a^	536.29 ± 39.48^a^	32.30 ± 1.62^a^	52.25 ± 3.48^a^
BSC2	35.62 ± 1.52^a^	101.96 ± 7.60^b^	363.95 ± 41.88^bc^	30.11 ± 1.31^a^	30.63 ± 1.02^b^
BSC3	30.95 ± 4.79^a^	91.74 ± 4.38^b^	283.25 ± 40.22^c^	34.90 ± 5.45^a^	32.10 ± 5.90^b^

The data are expressed as mean ± standard error (M±SE). The same column means no significant difference in the same line (Duncan’s multiple range tests, *p* < 0.01). UNF represents nutrient flux per unit area, NTR denotes nutrient translocation rate, NTC represents nutrient translocation capacity, UAF indicates active transport flow of nutrient, and NAC denotes nutrient active translocation capacity.

### 3.7. Se and Cd transport and enrichment in various organs of *C.* *violifolia*

**Figure 5.**
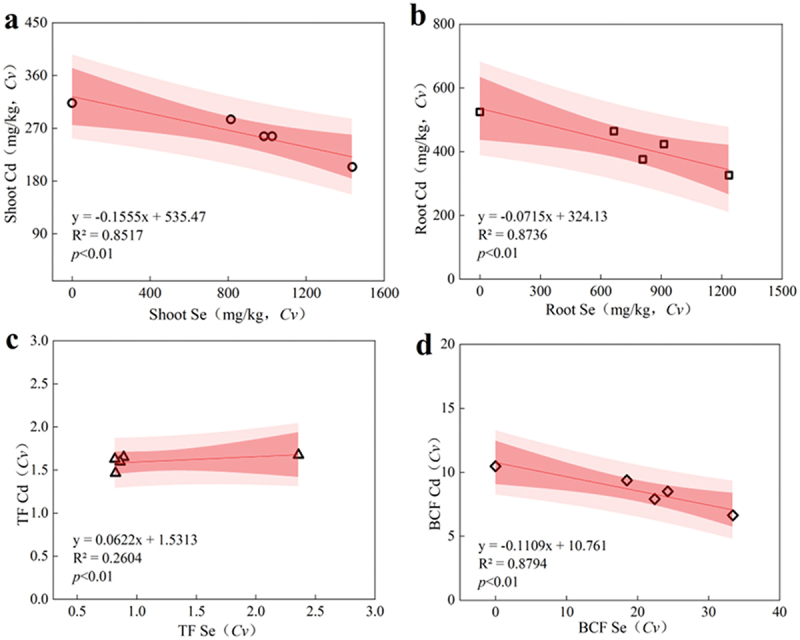
Se and Cd transport and enrichment in various organs. (a) correlation between total cadmium and selenium content of root, (b) correlation between total cadmium and selenium content of shoot, (c) translocation factor (TF) between total cadmium and selenium content, (d) bioconcentration factor (BCF) between total cadmium and selenium content.

Figure 5 and Table 6 demonstrated the total Se, total Cd content, Se-Cd translocation factor (TF Se and TF Cd) and bioconcentration factor (BCF Se and BCF Cd) of Se^6 +^ and Cd^2 +^ in different organs of *C. violifolia*. In comparison to CK, SC exhibited a significant deceased in the total Cd content. Conversely, BSC1 resulted in a maximum reduction in comparison to SC, whereas BSC3 led to a significant increase in comparison to SC. In comparison to SC, BSC1 resulted in a maximum increase, whereas BSC3 led to a maximum decrease in the total Se content.The total Se and Cd content in *C. violifolia* showed a significant negative correlation. The Se content in the shoots and roots ranged from 661.70 to 1436.62 mg/kg and from 811.91 to 1236.09 mg/kg, respectively, whereas the Cd content ranged from 204.33 to 313.20 mg/kg in the shoots and from 326.08 to 524.53 mg/kg in the roots, with R^2^ values of 0.87 and 0.85, respectively. The TF Se and TF Cd of *C. violifolia* showed no significant correlation, with R^2^ values of 0.26. TF Se was approximately 0.85, whereas TF Cd ranged from 1.46 to 1.70. BCF Se of *C. violifolia* showed a significant negative correlation, with an R^2^ of 0.88, with BCF Se ranging from 3 to 18, and BCF Cd ranging from 6 to 11.

**Table 6.** The content of Se and Cd in *C. violifolia.*

	CK	SC	BSC1	BSC2	BSC3
Shoot Cd	524.53 ± 2.16a	423.33 ± 3.57c	326.08 ± 2.00e	375.71 ± 3.13d	464.35 ± 1.56b
Root Cd	313.20 ± 2.94a	256.00 ± 1.45c	204.33 ± 2.85e	256.50 ± 1.96d	285.26 ± 3.62b
Shoot Se	＜0.02	913.34 ± 36.44b	1236.09 ± 23.31a	808.60 ± 4.97c	664.84 ± 3.66d
Root Se	＜0.02	1025.80 ± 25.86b	1436.62 ± 11.30a	983.59 ± 2.81c	814.07 ± 2.16d

The data are expressed as mean ± standard error (M±SE). The same column means no significant difference in the same line (Duncan’s multiple range tests, *p* < 0.01).

## 4. Discussion

### 4.1. Cd translocation influenced by the joint interactions of HCO_3_^−^and Se^6+^ versa growth

In this study, 1 mm HCO_3_^−^ and 0.46 mm Se^6+^ (BSC1) synergistically promoted *C. violifolia* growth, whereas 15 mm HCO_3_^−^ and 0.46 mm Se^6 +^ (BSC3) inhibited *C. violifolia* growth. The growth of *C. violifolia* at BSC1 attributed to the low HCO₃^−^ concentration, which reduced the bioavailability of Cd^2 +^ by increasing the pH of the root.^23^ It has been reported that the Cd^2 +^ present in environment including ion-exchangeable, oxidized, reduced, and residual states,^6^ and the ion-exchangeable Cd^2 +^ can directly harm plant growth, whereas HCO₃^−^ may reduce the exchangeable Cd^2 +^ in plant species by increasing the proportion of residual state Cd^2 +^, so as to decrease the bioavailability of Cd^2 +^ and make *C. violifolia* growth.^27^ In addition, BSC3 inhibited *C. violifolia* growth attributed to the simultaneous suppression of high HCO_3_^−^ and Cd^2 +^ in *C. violifolia*. As we know high HCO_3_^−^ supply and Cd^2+^ can inhibit plant growth reduced photosynthesis, impaired stomatal movement, and decreased water use efficiency.^28,29^

In this study, we hypothesized the synergistic effects of HCO₃^−^ and Se^6 +^ on the Cd^2+^ passivation. We found low HCO_3_^−^ supply and Se^6+^ synergistically promoted *C. violifolia* growth, whereas high HCO_3_^−^supply synergistically inhibited *C. violifolia* growth. In previous research, it was showed that HCO_3_^−^ can have positive and negative effects on plant growth. On the one hand, high HCO_3_^−^ supply reduced nutrient uptake, photosynthesis, leading to other inhibitions.^30^ Otherwise, In karst areas, low HCO_3_^−^ supply can serve as carbon source, enhancing photosynthesis and plants’ growth,^28,31^ provide energy for active transport. High HCO_3_^−^ supply reduce photosynthesis and energy. For example, at same HCO_3_^−^, *Euphorbia lathyris L*.(*El*) had the highest rate of HCO₃^−^ utilization, compared to *Orychophragmus violaceus L*. (*Ov*) and *Brassica juncea L*. (*Bj*), the highest bicarbonate use proportion was reached by *El*.^31^ Hence, it confirmed that low HCO_3_^−^ supply facilitated, whereas high HCO_3_^−^ supply hindered the Cd^2+^ passivation and, consequently, *C. violifolia* growth.

### 4.2. The antagonistic effect of selenium-cadmium in *C.* *violifolia*

It has been shown that all plant life activities involve charge separation, electron movement, and the transport of dielectric materials,^32^ the cell membranes of plants’ leaves changed ions, ionic groups, and electric dipoles to response the environments, attributed the changes of plant electrophysiological signals. In recent studies, plant electrophysiological dynamic parameters have been successfully applied to water and nutrient dynamics,^20,21,24^ the synergistic effects of HCO_3_^−^ and Se^6+^ on Cd^2+^ transport.^25^

Many studies have demonstrated that Se exhibits an antagonistic effect on the absorption and transport of Cd. ^11,12^ In this study, we found that the addition of Se^6+^ significantly reduced the Cd^2+^ content of *C. violifolia*, and significantly increased the ICP, IWHC, WTR, NTR, NTC, and NAC, but decreased the IR, IZ, IXc, and IX_L_ .

**Figure 6.**
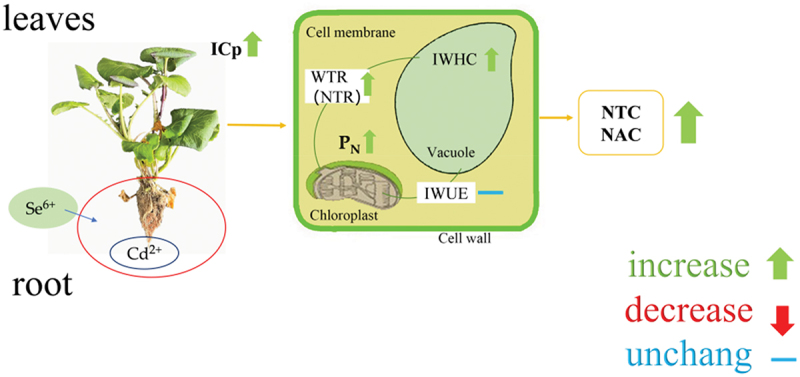
Se and Cd antagonistic mechanism. *P*_*N*_ represents the net photosynthetic rate, ICP indicates intrinsic capacitance, NTC denotes nutrient translocation capacity, NAC denotes nutrient active translocation capacity. IWHC indicates water-holding capacity, IWUE indicates intracellular water-use efficiency, WTR(NTR) denotes water(nutrient) translocation rate.

During vigorous plant growth and metabolic activities, a larger C corresponds to smaller values of R, Z, Xc, and X_L_,^33^ and the greater C, the stronger the cellular metabolic activity, the higher the IWHC, WTR, NTR, NTC, and NAC values, the stronger the active transport and nutrient transport capabilities.^20,21^ Therefore, the increase in selenium content and the decrease in cadmium content are attributed to high ICP, IWHC, WTR, NTR, NTC, and NAC and low IR, IZ, IXc, and IX_L_ because the translocation of selenium is active, while the translocation of cadmium is passive (Figure 6).

### 4.3. Intracellular water and nutrient dynamics characterizes the synergistic effect of HCO_3_^−^ and Se ^6+^ on Cd translocation in *C.* *violifolia*

**Figure 7.**
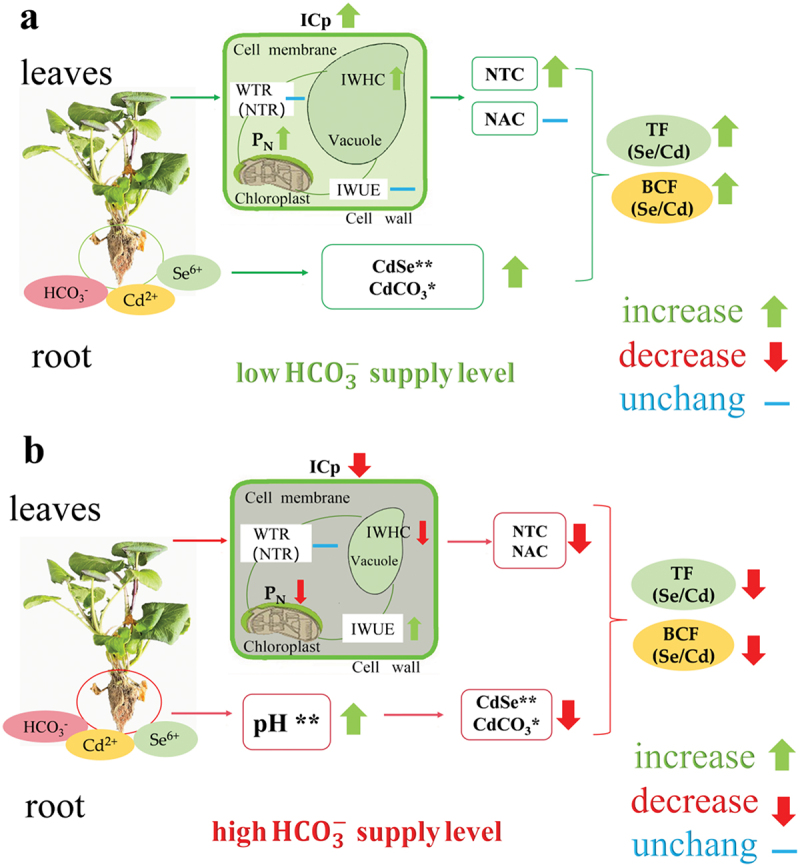
Mechanism of synergistic effect of HCO^−^_3_ and Se^6+^ on translocation of Cd^2+^ in *C. violifolia. P*_*N*_ represents the net photosynthetic rate, ICP indicates intrinsic capacitance, NTC denotes nutrient translocation capacity, NAC denotes nutrient active translocation capacity. IWHC indicates water-holding capacity, IWUE indicates intracellular water-use efficiency, and WTR(NTR) denotes water(nutrient) rate translocation.

In this study, we found the ICP, IWHC, WTR, NTR, NTC, and NAC was highest, and the IR, IZ, IXc, and IX_L_ were lowest, whereas the the total Cd content was lowest and the the total Se content were highest at BSC1. This indicates that low HCO_3_^−^ supply can enhance photosynthesis, plants’ growth and cellular metabolic activity by increasing selenium absorption and synergistically reducing the transport of cadmium. In BSC1, IWHC, WTR, NTR, NTC, and NAC can be attributed to the reduction of Cd^2 +^ of root, which improved water and nutrient translocation capacity of *C. violifolia* (Table 4, Table 5, Figure 7a). In addition, BSC1 increased water translocation capability by regulating stomatal opening and closing, as well as transpiration, to minimize water loss (Figure 2).^34^ Simultaneously due to low HCO_3_^−^ supply promoted growth and nutrient accumulation in *C. violifolia* (Tables 3, 5), which facilitated the formation of binding protein in cell membrane and the active transport,^35^ and decreased Cd^2+^ translocation in cells.^36^ It suggested that 1 mm HCO_3_^−^ enhance selenium’s antagonistic ability against cadmium, thereby reducing the mobility of cadmium in *C. violifolia* (Figure 7b).

Conversely, we found the ICP, IWHC, WTR, NTR, NTC, and NAC to be at its nadir, with the IR, IZ, IXc, IXL and IWUE reaching their highest values at BSC3. This indicated that the high HCO_3_^−^ (15 mm) supply surpassed the HCO_3_^−^ tolerance, leading to a negative effects on photosynthetic capacity, which affected nutrient and water translocation capacity, restricted stomatal movement in *C. violifolia* leaves.^22,37^ This indicated that high HCO_3_^−^ supply can suppress cellular metabolic activity by reducing selenium absorption and increasing cadmium translocation.

### 4.4. Synergistic effects of HCO_3_^−^ and Se^6+^on Cd translocation in *C.* *violifolia*

It has shown that Se^6 +^ -Cd^2 +^ has an antagonistic relationship, which means that Se^6 +^ inhibits the uptake and translocation of Cd^2 +^ in plant species.^14^ In this study, we found total Se, total Cd, and BCF in the roots were significantly negatively correlated (R^2^ >0.8), indicated a strong Se^6 +^ -Cd^2 +^ antagonistic relationship. However, there was no significant correlation (R^2^ <0.3) for TF(Se-Cd), suggesting that HCO_3_^−^ did not significantly affect the transport of Cd^2 +^ in *C. violifolia*.

In this study, we observed the total Se and the total Cd exhibited a significant negative correlation (Figure 7a and 7b), and the weak correlation for TF(Se-Cd). Therefore, we inferred the HCO_3_^−^ mainly only involved antagonism in root of *C. violifolia*. Bicarbonate can enhance the pH of the root,^5^ which made Cd^2+^ reduce in alkaline conditions to inhibit Cd^2+^ transport from root to shoot in *C. violifolia*.^38^ Moreover, we found BSC1 could promote the optimal growth of *C. violifolia*, due to the synergistic interaction of 1 mm HCO_3_^−^ and 0.46 mm Se^6 +^. In *C. violifolia* root, the Se^6+^ and Cd^2+^ predominantly combine to form Cd-Se, while a negligible HCO₃^−^ and Cd^2+^ formed CdCO₃. Besides, BSC1 can promote photosynthesis in *C. violifolia*, increase IWHC, NTC, and NAC, resulting in a significant enhancement of the active transport capacity of selenium, effectively preventing Cd^2+^ from entering the stem and leaves of *C. violifolia*. Compared to the plant species *Orychophragmus violaceus(Ov)*, which is adapted to karst environments, lower HCO₃^−^ concentrations (1 mm) effectively enhance the antagonism between selenium and cadmium in *C. violifolia*, as *Ov* has a greater ability to utilize bicarbonate.^25,31^

High HCO_3_^−^ supply was detrimental to its photosynthesis and growth.^37^ Therefore, the 15 mm HCO_3_^−^ inhibited of *C. violifolia* growth. Importantly, we found a significant negative correlation between BCF Se and BCF Cd, so was the TF Se and TF Cd in *C. violifolia*, which suggested that high concentrations of bicarbonates can counteract the antagonistic effect of selenium on cadmium of *C. violifolia*.

## 5. Conclusion

In this study, we found the joint effects of HCO_3_^−^ and Se^6+^on Cd^2+^ translocation in *C. violifolia*. BSC1 promoted growth in *C. violifolia*, whereas BSC3 inhibited. BSC1 synergistically antagonized Cd^2+^, it showed the greatest biomass and photosynthesis, attributed to the promotion of active translocation of Se^6+^ by low HCO_3_^−^ supply, manifesting a significant increase in IWHC, NTC, NAC, and decreased Cd^2+^ translocation. In contrast, BSC3 of *C. violifolia* had the smallest biomass and photosynthesis, attributed to high HCO_3_^−^ supply inhibited active translocation of Se^6+^, manifesting a significant decrease in NTC and NAC. Furthermore, no significant correlation was identified between TF Se and TF Cd in *C. violifolia*, indicating that HCO_3_^−^ may not participate the translocation of Cd^2+^ in *C. violifolia*, but may enhance pH of *C. violifolia* root to inhibit the Cd^2+^ from root to shoot of *C. violifolia*. This suggests that Se^6+^ instead of HCO_3_^−^ may play a primary role inhibiting Cd^2+^ in *C. violifolia*. Hence, this study offers a novel perspective for elucidating the joint interactions of HCO_3_^−^- Se^6+^ on Cd^2+^ transport in hyperaccumulator selenium plants species under karst areas.

## Abbreviations

C: capacitance
R: resisttance
Z: impedance
X_C_: capacitive reactance
X_L_: inductive reactance
IR: intrinsic resistance
IZ: intrinsic impedance
IXc: intrinsic capacitive reactance
IX_L_: intrinsic inductive reactanc
ICP: intrinsic capacitance
IWHC: intracellular water holding capacity
IWHT: intracellular water holding time
IWUE: intracellular water use efficiency
WTR: water transfer rate
UNF: nutrient flux per unit area
UAF: active transport flow of nutrient
NTC: nutrient translocation capacity
NAC: nutrient active translocation capacity
NTR: nutrient translocation rate
TF: translocation factor
BCF: bioconcentration factor
